# Sources of Blood Lead in Children

**DOI:** 10.1289/ehp.114-1332675

**Published:** 2006-01

**Authors:** Mary Jean Brown, David E. Jacobs

**Affiliations:** Lead Poisoning Prevention Branch, Centers for Disease Control and Prevention, Atlanta, Georgia, E-mail: mjb5@cdc.gov; U. S. Department of Housing and Urban Development, Washington, DC

In their article on seasonality and children’s blood lead (BPb) levels, Laidlaw et al. (2005) stated that “lead-contaminated soil in and of itself may be the primary driving mechanism of child BPb poisoning in the urban environment.” We believe that the data presented by Laidlaw et al. (2005) do not support this conclusion and that they misrepresent the many other studies of childhood lead poisoning, which support a more comprehensive, validated approach.

To support their “soil-only” hypothesis, Laidlaw et al. (2005) made three primary arguments: *a*) soil lead represents a large and available reservoir of environmental lead; *b*) resuspension of lead from contaminated soil followed by inhalation of airborne particulate matter < 10 μm in diameter (PM_10_) and dust deposition on interior surfaces is the major source of lead exposure to children; and *c*) the major source of lead contaminated soil is fallout from the past use of tetraethyl lead in gasoline.

Laidlaw et al. (2005) did not cite the compelling body of scientific evidence demonstrating that deteriorated lead-based paint and the contaminated dust and soil it generates is highly correlated with BPb levels in children. These have been reviewed at length elsewhere (National Academy of Sciences 1993; Jacobs 1995; President’s Task Force on Environmental Health Risks and Safety Risks to Children 2000). Indeed, Laidlaw et al. failed to recognize the enlightened statutory definition of the term “lead-based paint hazard,” which includes not only deteriorated lead-based paint but also interior settled house dust and bare soil. Together, these constitute the principal exposure sources and pathways for most (but not all) children today (Residential Lead-Based Paint Hazard Reducation Act of 1992—Title X 1992). Furthermore, documented evidence shows that soil lead levels are highest in soil at the house drip line and greatly decrease farther away from the house, regardless of whether or not the house is in a rural area or city (Jacobs 1995).

Laidlaw et al. (2005) ignored confounding due to the coexistence of old, poorly maintained lead-painted housing and traffic congestion in urban areas. They failed to develop any rationale to exclude lead paint as a prominent source of lead exposure and should have included a measure of it in their models. Furthermore, they did not support their assumption that PM_10_ data can be used as a surrogate for airborne lead particulate. Laidlaw et al. should have used the more direct measures of airborne lead particulate levels, which are available from the U.S. Environmental Protection Agency’s (EPA) National Ambient Air Quality program (U.S. EPA 2004), rather than the convoluted indirect measures of particulate matter < 10 μm in diameter (PM_10_), soil moisture, and other variables.

Studies of the effectiveness of soil removal in urban residential areas without addressing deteriorated lead paint have demonstrated that the “soil-only” approach being recommended by Laidlaw et al. (2005) is of limited value (U.S. EPA 1996). Even in Superfund sites where old mining and smelter wastes have resulted in very high soil lead levels, efforts that do not also address deteriorated lead paint often are disappointing. Furthermore, in the largest and most recent study of lead-based paint hazard control (which addressed lead paint hazards in > 3,000 homes in a dozen jurisdictions), house dust lead levels remained below preintervention levels for at least 3 years following the intervention (National Center for Healthy Housing and University of Cincinnati 2004). In a smaller follow-up study, dust lead levels remained between 11% and 75% lower than baseline levels for 6 years following lead-based paint hazard intervention (Wilson J, Pivetz T, Ashley P, Jacobs D, Strauss W, Menkedick J, et al., unpublished data). If the contention of Laidlaw et al. (2005) is correct (i.e., that urban soil lead is being resuspended and deposited inside homes), dust lead levels should have increased after intervention in these studies. In fact, they did not. This directly contradicts the authors’ conclusions.

Finally, Laidlaw et al. (2005) erroneously cited a pooled analysis (Lanphear et al. 1998), which they believe supports their view that soil and dust lead are the most significant predictors of children’s BPb. In fact, the model used in that study also included paint lead and paint condition as variables. If the dust and soil lead terms are forced out of the model, paint lead becomes the most significant predictor, which is consistent with the now well-known pathway of paint to settled house dust and bare soil, to children’s hands, to ingestion through hand-to-mouth contact. The pooled analysis (co-authored by D.E.J.) cannot be used to justify Laidlaw et al.’s “soil-only” approach.

The latest figures from the National Health and Nutrition Examination Survey indicate that the enormous disparity in the prevalence of BPb levels > 10 μg/dL once seen between African-American and white children has diminished greatly [Centers for Disease Control and Prevention (CDC) 2005]. Overall, the number of children in the United States with excessive BPb levels has declined from 890,000 in 1991–1994 to 310,000 in 1999–2002. Much of this is the result of federal, state, and local efforts to create a reservoir of lead-safe housing in communities at greatest risk. This success is tempered by recent evidence that a safe BPb level for children has not been demonstrated. The lack of a safe threshold reinforces the realization that to prevent the adverse health effects caused by lead exposure, we must exercise the wisdom to recognize and address the many sources of lead in children’s environments. The reality is too complicated and the cost of failure too devastating to reduce this to a one-source solution.
